# Selecting the species to be used in lichen transplant surveys of air pollution in Tunisia

**DOI:** 10.1007/s10661-023-11219-4

**Published:** 2023-04-14

**Authors:** Nadia Chahloul, Ayda Khadhri, Andrea Vannini, Mohamed Mendili, Aly Raies, Stefano Loppi

**Affiliations:** 1 Laboratory of Active Microorganisms and Biomolecules (LMBA), Faculty of Sciences of Tunis, University Tunis El-Manar-II, Tunis, Tunisia; 2 Plant, Soil, Environment Interactions Laboratory, Department of Biology, Faculty of Sciences, University of Tunis El-Manar II, Tunis, Tunisia; 3 Department of Life Sciences, University of Siena, Siena, Italy

**Keywords:** Air pollution, Bioaccumulation, Biomonitoring, Chlorophyll, PTEs, *Evernia prunastri*

## Abstract

This study was undertaken with the aim of selecting one or more lichen species that are the most suitable for transplant-based surveys of air pollution in Tunisia, in areas where the local native lichen vegetation is scanty or missing at all. To this purpose, four epiphytic (tree inhabiting) lichen species (*Evernia prunastri*, *Flavoparmelia caperata*, *Parmotrema perlatum*, *Ramalina farinacea*) were collected from the Babouch forests, a remote and unpolluted area of NW Tunisia, and analyzed for their content of potentially toxic elements (PTEs), namely Al, As, Cd, Cr, Cu, Fe, Ni, Pb, Sb, and Zn, by ICP-MS. Moreover, also the physiological status of the lichen samples was evaluated by measuring their chlorophyll content, photosynthetic efficiency, and spectral reflectance. The results indicated a remarkable contribution of airborne soil and dust particles to the total PTE content, especially for the foliose species *F. caperata* and *P. perlatum*. The fruticose lichens *E. prunastri* and *R. farinacea* had a lower and similar content of PTEs, and hence were regarded as more suitable to be used in transplant studies, since are able to detect even minimal accumulation amounts. All lichen species were healthy, as emerged from the analysis of physiological parameters.

**Supplementary Information:**

The online version contains supplementary material available at 10.1007/s10661-023-11219-4.

## Introduction

Air pollution is a major concern worldwide and has reached high levels of concern throughout the northern hemisphere, as well as a significant part of the southern hemisphere (Sicard, 2007). The release of substances of natural or anthropogenic origin is one of the most important factors in the degradation of the biosphere by humans. Indeed, large quantities of chemicals are released into the environment, most of which are considered hazardous. The introduction of these compounds implies serious risks for the environment and for human health. Examples of such compounds include potentially toxic elements (PTEs) that accumulate in the soil, the atmosphere, and the hydrosphere, disrupting the functioning of most ecosystems (Ramade, 1995), and are also known to be directly linked to the development of several diseases of the respiratory and digestive systems and other important pathologies (Singh & Prasad, 2015).

The current and future contamination of the environment requires a growing effort in experimental research. Biological indicators (bioindicators) can provide us with very interesting data on the levels, availability, deposition pattern, and biological effects of several pollutants (Berrayah, 2006). Among living organisms, lichens are excellent bioindicators for assessing the levels of pollution in the atmosphere (Loppi, 2014). Their use for depicting the deposition of airborne PTEs is well established (Contardo et al., 2020; Vannini et al., 2019), and there is evidence that lichens can also be profitably used to monitor the deposition of airborne microplastics (Loppi et al., 2021).

In recent times, the lichen transplantation technique, i.e., exposing lichen samples collected in a remote area into the study area of concern, has become very popular since it overcomes the problem of finding enough lichens in polluted areas; moreover, the initial elemental concentration and the exposure length are known (Loppi et al., 2019).

In Tunisia, research on the use of lichens to assess air quality is limited (Chahloul et al., 2022; Dhaouadi et al., 2022), and there is the need to establish the species or a set of species that can accumulate high levels of PTEs and, at the same time, are tolerant of their toxic effects. This/these species can be regarded as a model for future transplant studies to assess the levels of atmospheric pollution in urban and industrialized areas of Tunisia.

To this purpose, we focused our research on the levels of PTEs measured in four common lichen species collected in the Babouch forests, an unpolluted remote area of NW Tunisia. We compared the results from this site with two other sites previously studied in Tunisia (Chahloul et al., 2022) and also with the data for the same lichen species from Italy (Bargagli, 1998).

## Materials and methods

### Sample collection

Samples of the most abundant epiphytic (tree inhabiting) lichen species, i.e., *Evernia prunastri* (L.) Ach., *Parmotrema perlatum* (Huds.) M. Choisy, *Flavoparmelia caperata* (L.) Hale, and *Ramalina farinacea* (L.) Ach. (see Fig. 1S, Supplementary material), were randomly collected from the Babouch forests in the Jendouba region, NW Tunisia (36°47′57″ N, 8°40′54″ E) in September 2022. The area is remote and air pollution is negligible, as confirmed also by the abundant presence of *Lobaria pulmonaria*, a species that is known for its high sensitivity to air pollutants (Paoli et al., 2020). The species selected are among the most widely used in biomonitoring surveys (Abas, 2021), with marked habit differences, *F. caperata* and *P. perlatum* being foliose species, and *E. prunastri* and *R. farinacea* fruticose species.

### Chemical analysis

The lichen samples were air-dried, cleaned from any extraneous material under a binocular microscope, and pulverized using mortar and pestle and liquid nitrogen. Approximately 250 mg of lichen powder was dissolved in 3 mL of 70% HNO_3_, 0.2 mL of 60% HF, and 0.5 mL of 30% H_2_O_2_ in a microwave digestion system (Milestone Ethos 900) at 280 °C and 55 bar (Loppi, 2014). The concentrations of selected PTEs, namely Al, As, Cd, Cr, Cu, Fe, Ni, Pb, Sb, and Zn, were determined by Inductively Coupled Plasma Mass Spectrometry (ICP-MS, Perkin Elmer NexION 350). The quality of analytical procedures was verified using procedural blanks and the certified material IAEA-336 “Lichen” (International Atomic Energy Agency, 1999), which indicated recoveries in the range of 93–105%. The results are expressed on a dry weight basis (mg/kg dw). For each species, five replicates were measured.

### Physiological response

Lichen vitality was checked by monitoring some important physiological parameters, namely chlorophyll content, photosynthetic efficiency, and spectral reflectance (Vannini et al., 2018). Ten replicates were measured for each sample.

#### Chlorophyll content

The total content of chlorophyll was determined using a chlorophyll content meter (CCM 30 Opti-Sciences, Hudson, NH, USA) which gives chlorophyll estimates based on the radiation reflectance/absorption by chlorophyll (Gitelson et al., 1999). This method, which gives accurate measurements, in line with those obtained with the dimethylsulfoxide extraction method with (Liu et al., 2019), is commonly used in lichen biomonitoring (Chahloul et al., 2022). The data are expressed as mg chlorophyll per m^2^ of biological matter (mg/m^2^ dw).

#### Photosynthetic efficiency

The photosynthetic efficiency was measured with a Plant Efficiency Analyser (Handy PEA, Hansatech Ltd, Norfolk, UK), which is commonly used in lichen biomonitoring surveys (Paoli et al., 2020). Samples were moisten, left in the dark for 10 min, and then illuminated for 1 s with a saturating excitation pulse (1800 μmol s^−1^ m^−2^) of red light (650 nm), and the emission of fluorescence was then recorded. The commonly used parameter Fv/Fm indicator of the maximum quantum yield of PSII was used (Paoli et al., 2020).

#### Spectral reflectance

The spectral reflectance of chlorophyll was evaluated using the normalized difference vegetation index (NDVI), which is commonly used in similar studies (Garty, 2001). Measurements were taken with the PlantPen NDVI 310 device (Photon System Instruments, Drásov, Czech Republic).

### Statistical analysis

The Shapiro–Wilk test (*p* < 0.05) was used to verify that the data approached a normal distribution. After having checked the homogeneity of variances with the Bartlett test (*p* < 0.05), the significance of differences (*p* < 0.05) between species was verified with one-way permutation analysis of variance (ANOVA), coupled with the Duncan test for post hoc comparisons. Correlations (*p* < 0.05) were checked using Pearson’s correlation coefficient. All statistical tests were run with the free software R (R Core Team, 2022).

## Results

Overall, foliose lichens, i.e., *F. caperata* and *P. perlatum*, always showed the highest concentrations for all PTEs (Table 1). Significant differences (*p* < 0.05) in the accumulation capacities among species emerged for several PTEs, i.e., Al, Cr, Cu, Fe, Pb, and Zn, while the concentrations of As, Cd, and Sb (ranges: 0.65–1.04, 0.11–0.29, 0.16–0.25, respectively) were quite similar across the investigated species. Strong positive correlations (range 0.54–0.99) emerged for all elements except Cu, that was correlated only with Zn, and partly Zn, which showed several non-significant correlations (Table 2).

**Table 1:** Concentration of PTEs (mean ± standard error, mg/kg dw) in different lichen species, along with *F* values from ANOVA (* = *p* < 0.05). Values followed by a different letter in a column are statistically (*p* < 0.05) different after Duncan’s test

	Al	As	Cd	Cr	Cu	Fe	Ni	Pb	Sb	Zn
Evernia prunastri	1620 ± 14^b^	0.68 ± 0.03	0.15 ± 0.05	1.8 ± 0.1^a^	8.9 ± 0.1^b^	1105 ± 21^a^	1.9 ± 0.1^a^	3.9 ± 0.2^b^	0.16 ± 0.01	29.2 ± 0.9^a^
Flavoparmelia caperata	2514 ± 18^c^	0.84 ± 0.02	0.29 ± 0.02	3.1 ± 0.1^b^	9.0 ± 0.2^bc^	2124 ± 29^b^	3.0 ± 0.1^c^	7.7 ± 0.1^c^	0.20 ± 0.01	37.2 ± 0.2^b^
Parmotrema perlatum	2795 ± 21^d^	1.04 ± 0.02	0.17 ± 0.04	4.1 ± 0.1^c^	9.8 ± 0.3^c^	2755 ± 46^c^	2.2 ± 0.1^b^	8.4 ± 0.1^d^	0.25 ± 0.01	41.4 ± 0.4^c^
Ramalina farinacea	1465 ± 20^a^	0.65 ± 0.03	0.11 ± 0.02	1.8 ± 0.1^a^	7.5 ± 0.2^a^	1112 ± 17^a^	1.6 ± 0.1^a^	3.7 ± 0.1^a^	0.23 ± 0.01	30.3 ± 0.5^a^
Anova F	868*	1.7	0.11	270*	23*	2233*	127*	529*	0.17	452*

**Table 2:** Pearson’s correlation coefficients among PTEs. Bold = *p* < 0.05

	Al	As	Cd	Cr	Cu	Fe	Ni	Pb	Sb	Zn
Al	1.000									
As	**0.871**	1.000								
Cd	**0.630**	**0.607**	1.000							
Cr	**0.919**	**0.989**	**0.637**	1.000						
Cu	0.493	0.123	−0.004	0.174	1.000					
Fe	**0.934**	**0.977**	**0.633**	**0.997**	0.192	1.000				
Ni	**0.992**	**0.916**	**0.655**	**0.956**	0.404	**0.967**	1.000			
Pb	**0.880**	**0.985**	**0.661**	**0.991**	0.065	**0.987**	**0.925**	1.000		
Sb	**0.544**	**0.868**	0.422	**0.825**	−0.298	**0.803**	**0.636**	**0.852**	1.000	
Zn	**0.765**	0.373	0.442	0.477	**0.776**	**0.518**	**0.706**	0.393	−0.032	1.000

Variations among lichen species in ecophysiological parameters were smaller (Table 3). The chlorophyll content was fairly similar across species (419–490 mg/m^2^), with the only exception of lower values (280 mg/m^2^) for *R. farinacea*. Also the photosynthetic efficiency was very similar (Fv/Fm in the range 0.75–0.77), with a slightly higher value (Fv/Fm = 0.80) for *F. caperata*. The spectral reflectance showed the same trend as PTEs, with similar, lower values for the fruticose species *R. farinacea* and *E. prunastri* (NDVI = 0.24) and higher values for the foliose species *F. caperata* and *P. perlatum* (NDVI in the range 0.35–0.47). The ecophysiological parameters were substantially unrelated to the content of PTEs (Table 4).

**Table 3:** Chlorophyll content (mg/m^2^), photosynthetic efficiency (Fv/Fm), and spectral reflectance (NDVI) in lichen samples (mean ± standard error), along with *F* values from ANOVA (* = *p* < 0.05). Values followed by a different letter in a column are statistically (*p* < 0.05) different after Duncan’s test

	Chlorophyll content	Photosynthetic efficiency	Spectral reflectance
Evernia prunastri	490 ± 115^b^	0.77 ± 0.01^ab^	0.24 ± 0.10^a^
Flavoparmelia caperata	493 ± 77^b^	0.80 ± 0.06^b^	0.47 ± 0.04^b^
Parmotrema perlatum	419 ± 120^b^	0.75 ± 0.05^a^	0.35 ± 0.06^b^
Ramalina farinacea	280 ± 101^a^	0.75 ± 0.04^a^	0.24 ± 0.05^a^
Anova F	7.4*	4,522*	22.5*

**Table 4:** Pearson’s correlation coefficients between PTEs and physiological parameters

	Al	As	Cd	Cr	Cu	Fe	Ni	Pb	Sb	Zn
Chlorophyll content	0.213	−0.040	0.164	0.019	0.295	0.040	0.165	0.022	−0.323	0.345
Photosynthetic efficiency	0.268	0.212	0.557	0.245	−0.126	0.258	0.277	0.290	0.099	0.199
Spectral reflectance	0.407	−0.004	0.405	0.124	0.502	0.172	0.353	0.063	−0.287	0.828

A comparison of the average concentrations of PTE measured in foliose and fruticose lichens from remote areas of NW Tunisia (Babouch, this study—Oued zeen and Mejen essef, Chahloul et al., 2022) with background values reported for foliose and fruticose lichens from Italy (Cecconi et al., 2019) is shown in Table 5. The concentrations were overall higher in Tunisia, especially for Al and Fe, and Tunisian foliose lichens showed the highest concentrations for all elements.

**Table 5:** Comparison of the content (mg/kg dw) of some PTEs in foliose and fruticose lichens from Italy (Cecconi et al., 2019) and Tunisia (present study)

	Al	As	Cd	Cr	Cu	Fe	Ni	Pb	Sb	Zn
Italy foliose lichen	253	0.18	0.18	1.2	6.2	-----	1.3	6.7	-----	48.2
Italy fruticose lichen	571	0.24	0.13	1.7	5.1	523	1.8	3.7	0.09	26.8
Tunisia foliose lichen	1696	0.93	0.22	3.7	14	1354	4.6	2.4	0.17	35.3
Tunisia fruticose lichen	1635	0.71	0.10	1.9	9.4	1172	1.8	2.4	0.14	22.5

## Discussion

The analysis of PTEs in the investigated lichen species showed that variations are largely species-specific, but with higher values for foliose species. Also Bargagli et al. (2002) found that the foliose *F. caperata* accumulates significantly higher concentrations of several PTEs such as Hg, Cd, Pb, Cu, V, and Zn, compared with the fruticose lichens. Also Kumar and Mishra (2014) concluded that foliose lichens are more effective in accumulating wet and dry atmospheric depositions and suggested that their morphology provides a wider surface for the deposition and absorption of pollutants.

Lichens accumulate both essential and non-essential elements by several mechanisms, e.g., surface complexation, biomineralization, and trapping of dust and soil particles in the intercellular spaces of the medulla (Rola et al., 2016). Moreover, the accumulation of elements in lichens is related both to the chemical properties of the element itself and the properties of the associated particles, especially solubility (Alami et al., 2014). Indeed, Al and Fe were accumulated in large quantities in all samples, and their concentrations were strongly correlated. These are clear indications of soil contamination of samples (Loppi et al., 2004), suggesting that a large part of the lichen elemental content may have originated from soil particles (Guevara et al., 1995). Moreover, Al and Fe are also common in airborne dust that can deposit over the lichen thallus (Garty, 2001). The large occurrence of elements trapped as particles explains the huge accumulation of PTEs found in lichens growing in polluted environments (Garty, 1987). Interestingly, the concentration of Cu was not correlated with those of Al and Fe and the other PTEs, suggesting a different origin for this element, possibly from Cu-based products largely used in agriculture in the areas surrounding Babouch.

The analysis of physiological parameters, beyond the small statistical differences, indicated an overall good vitality of samples, and a lack of correlation with the content of PTEs, further confirming that air pollution in the study area in negligible. Values of Fv/Fm, the common indicator of photosynthetic efficiency, in healthy lichens are usually around 0.7, with values < 0.6 being indicative of some stress (Nayaka et al., 2009). All our samples showed values > 0.75, clearly indicating an efficient quantum yield of photosystem II. The small differences emerged between species can be easily explained by the fact that shade- and sun-exposed lichens may have slightly different photosynthetic performances (Baruffo & Tretiach, 2007; Vannini et al., 2017). Also the content of chlorophyll, which is commonly used as an indicator of lichen vitality (Von Arb et al., 1990) found in our lichens, is indicative of unpolluted environments (González & Pignata, 1997). The differences found (i.e., smaller values in *R. farinacea*) are due to interspecific differences, and the effect of site-related micro-environmental parameters (Cercasov et al., 2002). It has been reported that low NDVI values are related to high levels of some air pollutants, and that this parameter is suitable for the detection of early stress indications before variations in other ecophysiological parameters become apparent (Garty, 2001). Our results showed lower NDVI values in fruticose lichens and this could be both due to intrinsic features and to a higher sensitivity to air pollutants, which is usually a characteristic of fruticose lichens (Hawksworth & McManus, 1989).

Some studies have noted a correlation between the growth form of the lichen and its bioaccumulation capacity, and have concluded that this functional trait is a key factor affecting the bioaccumulation of PTEs in lichens (Chiarenzelli et al., 1997; Bačkor & Loppi, 2009). Our results showed that Tunisian lichens have higher concentrations of most elements compared with Italian lichens, especially for Al and Fe, and that foliose Tunisian lichens have the highest concentrations for all the investigated PTEs. This is much likely due to the higher accumulation of airborne soil and dust particles, but also to the fact that the foliose growth form has a more extensive and intimate physical contact with the bark substrate through the rhizinae, which allows for a more efficient trapping of elements not or less dissolved in the rainfall flowing down the tree trunk (Brown et al., 1994).

On the other side, the fruticose growth form has a much more limited contact with the substrate, usually a single point of attachment. These features are known to largely influence element uptake, which may differ markedly between foliose and fruticose species (St. Clair et al., 2002).

## Conclusions

This study was undertaken with the aim of selecting one or more lichen species that are the most suitable for transplant-based surveys of air pollution in Tunisia, in areas where the local native lichen vegetation is scanty or missing at all. The results indicated that the fruticose lichens *E. prunastri* and *R. farinacea* have a lower and similar content of PTEs, and hence are suitable to detect even subtle accumulation amounts. These species are also healthy, as emerged from the analysis of physiological parameters. In case just one species has to be selected, it is suggested that *E. prunastri* be used since this species has a higher chlorophyll content that can be useful to detect even small decreasing in this pigment.

## Supplementary Information

Below is the link to the electronic supplementary material.

Supplementary file1 (DOC 1582 kb)

## Data Availability

The row data are available on request from the corresponding author.
