# Supplementary material for: Selecting the species to be used in lichen transplant surveys of air pollution in Tunisia
Source: Environ Monit Assess. 2023 Apr 14;195(5):570. doi: 10.1007/s10661-023-11219-4 (PMC10104911; doi:10.1007/s10661-023-11219-4)
Supplement: Supplementary file 1 — Supplementary file1 (DOC 1582 kb) [file 10661_2023_11219_MOESM1_ESM.doc]

**
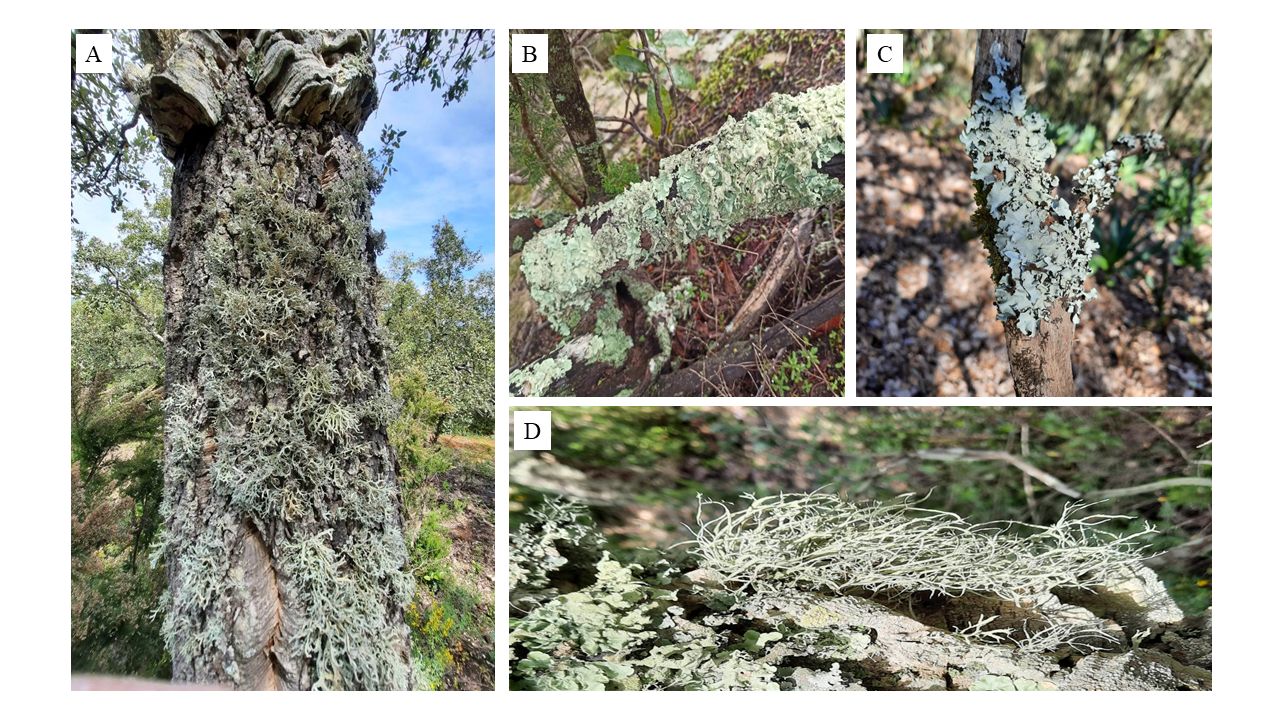
**

**Figure 1S**

The four lichen specie sampled:

A = Evernia prunastri

B = Flavoparmelia caperata

C = Parmotrema perlatum

D = Ramalina farinacea
